# Deficits in coordinated neuronal activity and network topology are striatal hallmarks in Huntington’s disease

**DOI:** 10.1186/s12915-020-00794-4

**Published:** 2020-05-28

**Authors:** S. Fernández-García, J. G. Orlandi, G. A. García-Díaz Barriga, M. J. Rodríguez, M. Masana, J. Soriano, J. Alberch

**Affiliations:** 1 Departament de Biomedicina, Facultat de Medicina, Institut de Neurociències, Universitat de Barcelona, 08036 Barcelona, Spain; 2 Institut d’Investigacions Biomèdiques August Pi i Sunyer (IDIBAPS), 08036 Barcelona, Spain; 3 Centro de Investigación Biomédica en Red sobre Enfermedades Neurodegenerativas (CIBERNED), 28031 Madrid, Spain; 4 Complexity Science Group, Department of Physics and Astronomy, Faculty of Science, University of Calgary, Calgary, AB T2N 1N4 Canada; 5 Departament de Física de la Matèria Condensada, Universitat de Barcelona, 08028 Barcelona, Spain; 6 Universitat de Barcelona Institute of Complex Systems (UBICS), 08028 Barcelona, Spain; 7 Production and Validation Center of Advanced Therapies (Creatio), Faculty of Medicine and Health Science, University of Barcelona, 08036 Barcelona, Spain

**Keywords:** Network, Striatum, Calcium imaging, Neuronal activity, Huntington’s disease

## Abstract

**Background:**

Network alterations underlying neurodegenerative diseases often precede symptoms and functional deficits. Thus, their early identification is central for improved prognosis. In Huntington’s disease (HD), the cortico-striatal networks, involved in motor function processing, are the most compromised neural substrate. However, whether the network alterations are intrinsic of the striatum or the cortex is not fully understood.

**Results:**

In order to identify early HD neural deficits, we characterized neuronal ensemble calcium activity and network topology of HD striatal and cortical cultures. We used large-scale calcium imaging combined with activity-based network inference analysis. We extracted collective activity events and inferred the topology of the neuronal network in cortical and striatal primary cultures from wild-type and R6/1 mouse model of HD. Striatal, but not cortical, HD networks displayed lower activity and a lessened ability to integrate information. GABA_A_ receptor blockade in healthy and HD striatal cultures generated similar coordinated ensemble activity and network topology, highlighting that the excitatory component of striatal system is spared in HD. Conversely, NMDA receptor activation increased individual neuronal activity while coordinated activity became highly variable and undefined. Interestingly, by boosting NMDA activity, we rectified striatal HD network alterations.

**Conclusions:**

Overall, our integrative approach highlights striatal defective network integration capacity as a major contributor of basal ganglia dysfunction in HD and suggests that increased excitatory drive may serve as a potential intervention. In addition, our work provides a valuable tool to evaluate in vitro network recovery after treatment intervention in basal ganglia disorders.

## Background

In neurodegenerative diseases, alterations in brain circuits often appear before any behavioral symptoms [1]. Recent advances in network neuroscience and imaging techniques have provided the tools to quantify these early-stage alterations. A common signature of disease-induced network disruption is the inability to develop or sustain coordinated activity, a general feature underlying the strength of a circuit and its capacity to transmit information [2]. Understanding intrinsic network alterations that propagate across circuits in neurodegenerative diseases is essential to slow their progression. Thus, new therapeutic approaches should focus on restoring network activity as soon as it is disrupted [3].

In Huntington’s disease (HD) in particular, cortico-striatal networks are the most vulnerable [4]. HD is an inherited neurodegenerative condition caused by the mutation of the huntingtin gene (mHtt) and characterized by a triad of symptoms including motor, cognitive, and psychiatric disturbances [5]. The most affected brain region in HD is the striatum, a prominent main hub of the basal ganglia circuit [6], which receives dense glutamatergic inputs from the cortex and plays a crucial role in processing information related to motor function, reward, and goal-oriented behavior [7]. In HD, striatal medium-sized spiny neurons (MSNs) receive unbalanced excitatory/inhibitory signaling that has been related to altered excitatory cortico-striatal activity [8, 9] and inhibitory activity within the striatum [10]. The presence of mHtt induces an increase in the striatal excitatory synaptic activity at early stages of the disease [11] followed by a progressive decrease, associated with a striatal disconnection from the cortex [12]. Furthermore, increased inhibitory synaptic activity occurs early in mouse models [13]. Moreover, mHtt generates reduced feedback inhibition and atypical bidirectional communication between pairs of MSNs [10]. However, how these effects of mHtt translate to alterations of communication in large-scale network dynamics are not fully understood.

Large-scale calcium imaging experiments with primary cultures have become a valuable tool for the study of network dynamics in a controlled environment [14]. Neuronal cultures are typically prepared from cells of specific brain regions. A characteristic feature of neuronal cultures is their potential to self-organize and produce coordinated ensemble activity that encompasses the entire network [15–17] or a subset of neurons [18, 19]. The analysis of this activity in the context of graph theory unravels neuronal organization principles at the microscale level, i.e., the topological configuration of the network [20], and exposes key differences between isolated healthy and diseased circuits.

Here, we evaluate the impact of mHtt on network dynamics in isolated striatal and cortical primary neuronal cultures derived from the R6/1 mouse model of HD. Among the different mouse models available (Menalled and Chesselet [21]), R6/1 mice are extensively used and are very well characterized, exhibiting an early onset of the disease and severe phenotype [21, 22]. Furthermore, previous work with R6/1 striatal primary cultures showed that HD-related deficits can be observed in their spontaneous neuronal activity [23]. In the present work, we provide evidence that striatal—but not cortical—circuits in HD display a network structure with high segregation that decreases its integration capacity. Moreover, challenging the striatal network uncovers the compromised information processing capabilities of the system. Finally, we found that by increasing the excitatory drive of the striatal system, we rectify a number of features of the altered network, highlighting a possible therapeutic target for early intervention in HD.

## Results

### Number of active neurons and their coordinated ensemble activity are reduced in striatal, but not cortical, HD mouse cultures

To study how mutant huntingtin is affecting the number of active neurons and their activity, we used primary culture of striatal or cortical neurons from wild-type (WT) or R6/1 mice (HD) at 15 days in vitro (DIV). We considered this timepoint as optimal since neurons are mature enough to show activity and the network is sufficiently developed and stable to exhibit rich collective dynamics in striatal cultures [23, 24]. Based on extensive literature on cortical cultures, earlier timepoints are too close to GABA-switch, and later timepoints (e.g., from 20 DIV onwards) are known to correspond to strong dynamical changes in the form of a decay in network bursting [25, 26]. Moreover, HD cultures at 15 DIV do not present altered morphology (Figure S1), as has been described in earlier timepoints [23].

**Fig. 1:**
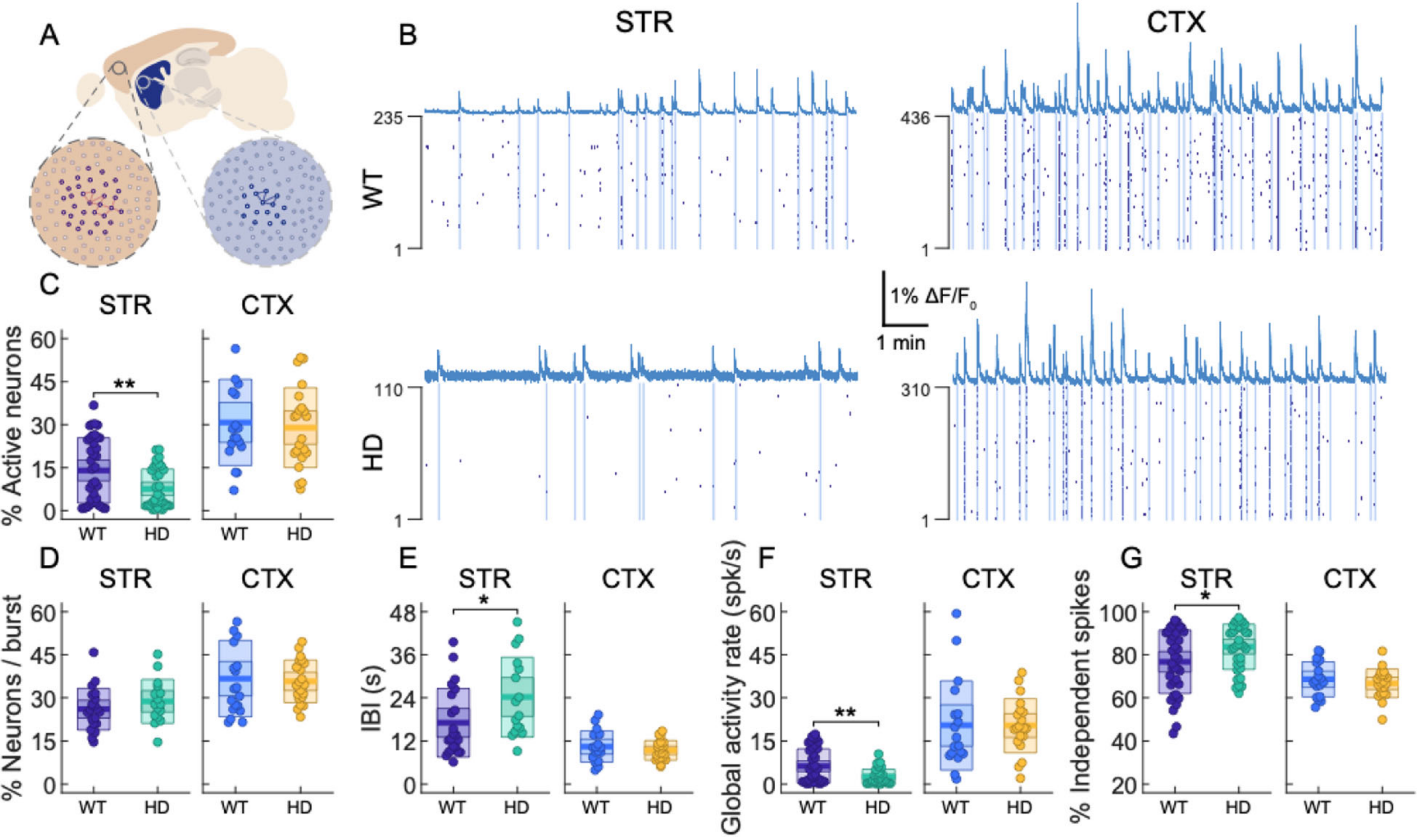
Spontaneously active neurons and coordinated ensemble activity were reduced specifically in the striatum, but not the cortex, of primary cultures from the R6/1 HD model (HD) compared to wild-type (WT). **a** Primary cultures were obtained from the striatum and cortex of E18 WT and HD mouse embryos, and neuronal spontaneous activity was measured using fluorescence calcium imaging at 15 DIV. **b** Representative average fluorescence trace and reconstructed spikes from neurons are represented in raster plots for WT (top) and HD (bottom) for striatal (STR, left) and cortical (CTX, right) cultures. Vertical blue bars highlight network bursts. **c** Percentage of active neurons in the cultures. **d** Percentage of active neurons that participate in spontaneous network burst. **e** Average network inter-burst interval (IBI), i.e., the average time between consecutive network bursts. **f** Global activity rate of the cultures, i.e., total number of spikes per unit time within the field of view. **g** Fraction of independent spikes, i.e., those spikes not followed or preceded by any other spike within 50 ms in the whole system. **c**–**g** Each circle in the plot represents a single culture from at least three different litters (**c**, **f**, **g**: STR WT *n* = 38; STR HD *n* = 37; CTX WT *n* = 19; CTX HD *n* = 22) (**d**, **e**: STR WT *n* = 22; STR HD *n* = 16; CTX WT *n* = 19; CTX HD *n* = 22), thick line the mean, thick shaded area the standard error of the mean, and thin shaded area the standard deviation. Statistical analysis was performed using two-sample Student’s *t* test between WT and HD populations, *p < 0.05, **p < 0.01

In these cultures, at least a 90% of cells were neurons, with a small proportion of astrocytes (Additional file 1: Figure S1). Moreover, we also investigated the morphology of the neurons by measuring the number of branching and total tree length as previously described [27]. We did not find any significant differences in the neuronal structure between genotypes at 15 DIV (Additional file 1: Figure S1). Then, we analyzed the spontaneous calcium mobilization events of ~ 1000 single cells and evaluate the dynamical traits of ensemble activity in cortical (CTX) and striatal (STR) primary cultures from WT and the HD model (Fig. 1a; Additional file 2: Figure S2, see the “Methods” section for further details). We used a combination of high-throughput calcium imaging and its analysis through the NETCAL software, which we developed in parallel to this study [28]. From the measured calcium transients, we reconstructed the spike trains for each neuron and used these “reconstructed spikes” (referred as “spikes” from here onwards) to quantify neuronal activity.

Striatal and cortical primary cultures showed spontaneous neuronal activity (Fig. 1b, c). In the case of HD, striatal cultures exhibited a decreased fraction of active neurons with respect to WT (WT, 14 ± 2%; HD, 8 ± 1%; *p* = 0.006) while the percentage of active neurons showed no significant differences between genotypes in cortical cultures (WT, 31 ± 4%; HD, 29 ± 3%; *p* = 0.7). Remarkably, the average fraction of active neurons participating in network bursts (large ensembles of coordinated activity, Fig. 1d) was similar between genotypes in both striatal and cortical cultures (STR: WT 26 ± 2%, HD 29 ± 2%, *p* = 0.3; CTX: WT 37 ± 3%, HD 36 ± 2%, *p* = 0.8). However, the inter-burst interval of network bursts (IBI), which is the most salient observable of collective activity in cultures [29], was significantly higher in HD striatal cultures than in the WT ones (WT, 17 ± 2 s; HD, 24 ± 3 s; *p* = 0.04; Fig. 1e), indicating that HD striatal cultures show less coordinated activity. Surprisingly, no differences were detected in cortical cultures (WT, 10 ± 1 s; HD, 9.4 ± 0.6 s; *p* = 0.4; Fig. 1e). Despite the variability observed across cultures, the effects observed were independent on the different litters, as intra- and inter-litter variability were of the same order (Additional file 3: Figure S3); therefore, we pooled all the litters under study together to avoid possible intra-litter bias. Additionally, network burst duration and burst amplitude (i.e., the number of spikes per neuron within a burst) were similar between genotypes in both striatal and cortical cultures, suggesting that the internal structure of these events is maintained between genotypes (Additional file 4: Figure S4). In addition, single-cell spontaneous activity did not show major differences across genotypes for striatal or cortical cultures (Additional file 5: Figure S5).

To further explore alterations in network dynamics, we evaluated the global activity of the cultures by measuring the global activity rate (Fig. 1f) and the fraction of independent spikes (Fig. 1g). The “global activity rate” measures the average number of total reconstructed spikes per unit time in the culture. The fraction of “independent spikes” within the system represents the fraction of reconstructed spikes that are more than 1 frame (50 ms) apart from any other spike in the system. Independent spikes are a proxy of synaptic efficacy, since they represent synaptic events that are isolated and do not participate in episodes of coordinated ensemble activity such as network bursts. Global activity rate was significantly lower in HD striatal cultures compared to WT, indicating that HD striatal cultures were overall less active (STR WT 6.3 ± 1.0 spikes/s vs STR HD 2.5 ± 0.4 spikes/s; *p* = 0.0013; Fig. 1f). Cortical cultures showed no significant differences in global activity rate (CTX WT 20 ± 4 spikes/s vs CTX HD 20 ± 2 spikes/s; *p* = 0.9). Moreover, the fraction of independent spikes was significantly higher in HD striatal cultures (STR WT 76 ± 2% vs STR HD 84 ± 2%; *p* = 0.02; Fig. 1g), and no differences were observed in cortical cultures (CTX WT 69 ± 2% vs CTX HD 67 ± 1%; *p* = 0.4). Overall, these results indicate that HD striatal, but not cortical, cultures present a larger fraction of isolated spikes, i.e., ones that do not trigger a response anywhere within the network, pointing towards reduced synaptic transmission in these cultures.

### Network analysis reveals a loss of efficiency and an increase in segregation of HD striatal cultures under basal conditions

**Fig. 2:**
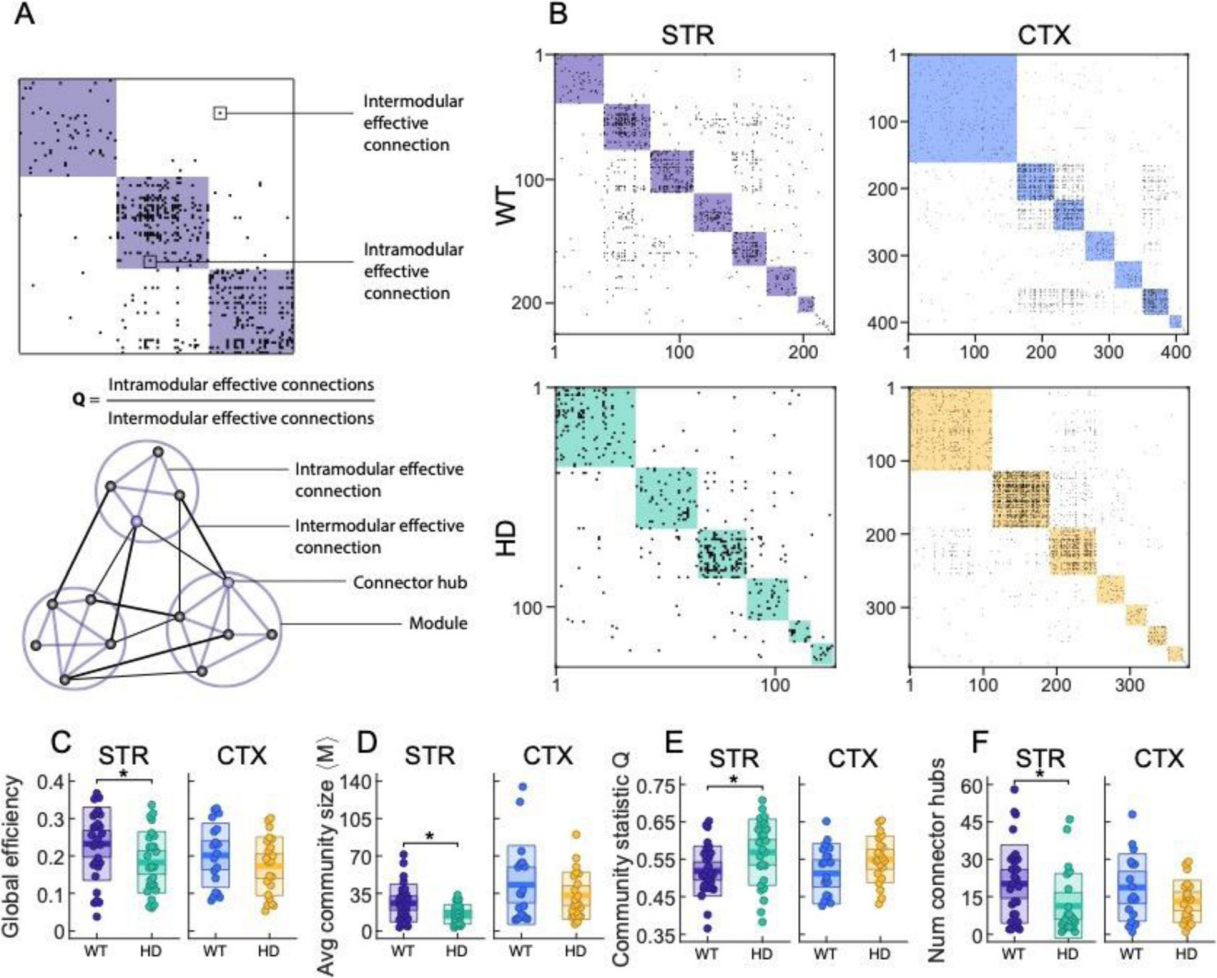
Network topology properties of striatal, but not cortical, cultures are altered in HD. **a** Representative effective connectivity matrix of a neuronal culture. Each dot represents an effective connection from neuron to neuron, i.e., neuron *i* → neuron *j*. Communities were identified using the Louvain community detection algorithm and are highlighted in color. Connections between neurons that belong to the same community (module) are intra-modular effective connections, while effective connections between neurons not belonging to the same community are inter-modular connections. **b** Representative effective connectivity matrices of WT and HD striatal and cortical neuronal cultures. **c**–**f** Network topology properties for WT and HD striatal and cortical cultures under basal conditions. **c** Global efficiency, i.e., average of the inverse shortest path length. **d** Average community size, i.e., average number of neurons per module. **e** Community statistic *Q*. **f** Average number of connector hubs, i.e., large neuronal nodes that connect mostly with nodes from other communities. Each circle represents a single experiment (STR WT *n* = 29; STR HD *n* = 24; CTX WT *n* = 19; CTX HD *n* = 22), thick line the mean, thick shaded area the standard error of the mean, and thin shaded area the standard deviation. Statistical analysis was performed using two-sample Student’s *t* test between WT and HD populations, *p < 0.05

The topological configuration of a network is directly associated to its local and global efficiency, which together determine the network’s capacity to integrate information effectively [30]. For this reason, effective network topologies were constructed using Generalized Transfer Entropy [31], a network inference method specifically developed to infer causal relations (in the Granger sense) between neurons in calcium imaging experiments. Effective connections between pairs of neurons are represented in effective connectivity matrices, which also display the organization of the network in communities (modules) (Fig. 2a). Communities were identified using the Louvain community detection algorithm [32], which subdivides networks into non-overlapping groups of neurons, maximizing the number of within-group connections (intra-modular effective connections), and minimizing the number of between-group connections (inter-modular effective connections). The statistic *Q* is the ratio between intra-modular and inter-modular effective connections and thus informs about the segregation of the neuronal network. We also measured the global efficiency of the network, which considers the number of steps (path length) necessary to route information throughout the network.

Figure 2b provides representative effective connectivity matrices of the two investigated culture types. Specifically, we observed decreased global efficiency between striatal HD and WT cultures (STR WT 0.23 ± 0.02 vs STR HD 0.18 ± 0.02; *p* = 0.04; Fig. 2c), indicating that HD striatal cultures have an average longer path length. These differences were not present in cortical cultures (CTX WT 0.20 ± 0.02 vs CTX HD 0.17 ± 0.02; *p* = 0.3). Analysis of network communities also revealed significant differences between WT and HD striatal cultures, with HD cultures exhibiting significantly smaller communities (STR WT 26 ± 3 vs STR HD 16 ± 2; *p* = 0.011; Fig. 2d) and a higher community statistic *Q* (STR WT 0.52 ± 0.01 vs STR HD 0.57 ± 0.02; *p* = 0.02; Fig. 2e). The higher *Q*, the weaker the coupling among communities. Thus, these results indicate that HD striatal cultures are more segregated, with proportionally less inter-modular connections. HD striatal cultures also showed a decreased number of connector hubs (STR WT 20 ± 3 vs STR HD 11 ± 3; *p* = 0.03; Fig. 2f). Connector hubs denote large degree nodes with a high participation coefficient, i.e., community nodes that connect with nodes from other communities. The low number of connector hubs in HD striatal cultures revealed weak inter-modular connectivity. Cortical cultures, however, did not show any statistical differences between genotypes on network community structure. Overall, network differences in striatal cultures (under basal conditions) point towards a deficit in information flow and routing on the HD-affected circuitry.

### Blockade of GABA_A_ receptors uncovers increased inhibition as a critical factor underlying striatal network alterations in HD cultures

**Fig. 3:**
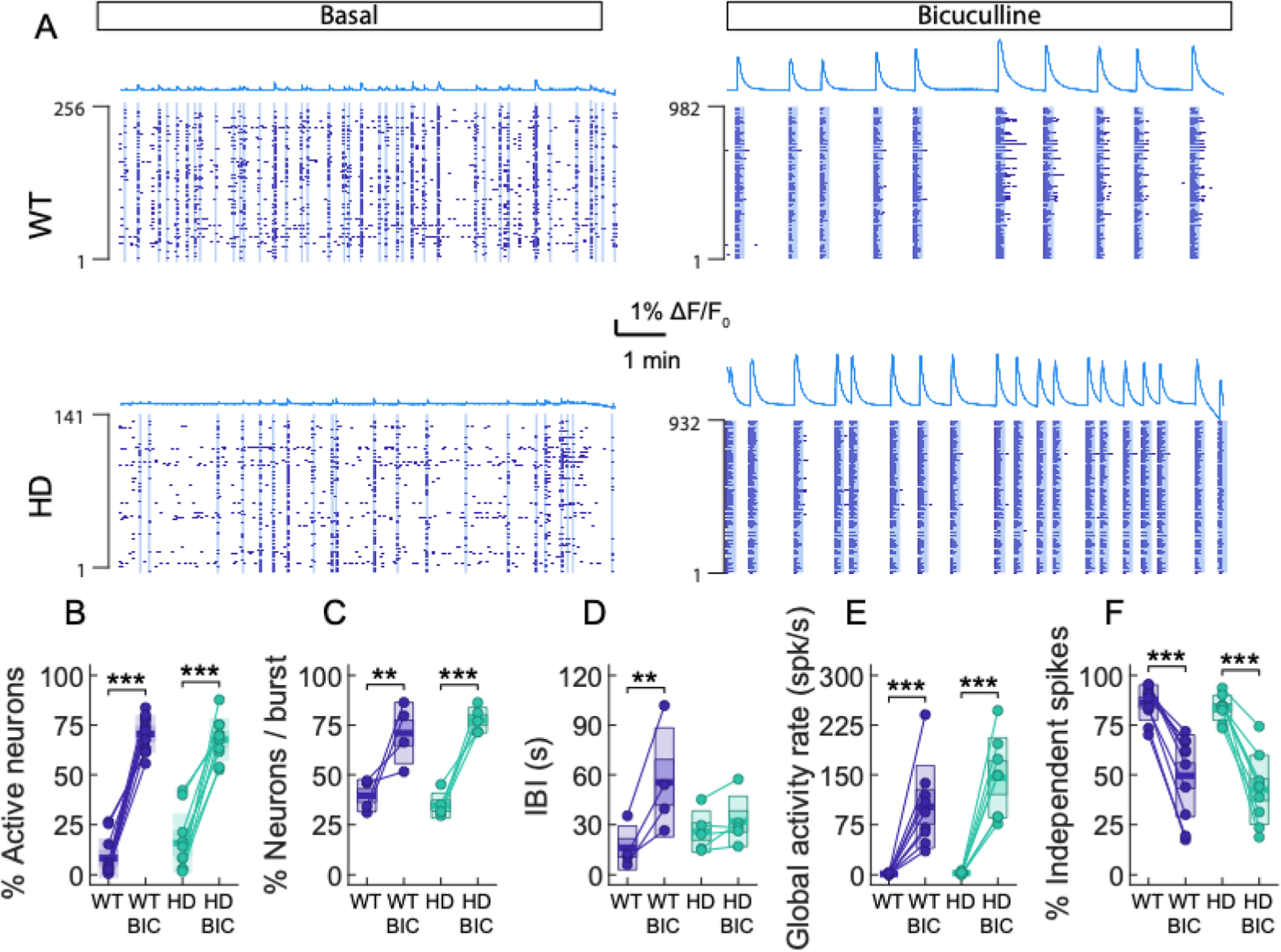
GABA_A_ receptor blockade with bicuculline (BIC) differentially induces network burst events in striatal cultures of WT or HD. **a** Spontaneous neuronal activity was recorded during 10 min in basal conditions and after addition of bicuculline (BIC, 60 μM). The plots show representative average fluorescence trace, and reconstructed spikes from neurons are represented in raster plots for WT (top) and HD (bottom) for baseline (left) and after system wide disinhibition with BIC 60 (right). Vertical blue bars highlight network bursts. **b** Percentage of active neurons before and after the addition of BIC. **c** Percentage of active neurons participating in network bursts before and after the addition of BIC and **d** average inter-burst interval (IBI). **e** Global activity rate of the cultures. **f** Fraction of independent spikes. Each circle represents a single experiment (**b**, **e**, **f**: STR WT *n* = 11; STR HD *n* = 10; CTX WT *n* = 19; CTX HD *n* = 22) (**c**, **d**: STR WT *n* = 4; STR HD *n* = 5; CTX WT *n* = 19; CTX HD *n* = 22), thick line the mean, thick shaded area the standard error of the mean, and thin shaded area the standard deviation. Statistical analysis was performed using mixed ANOVA and posterior post hoc test with Bonferroni’s multiple comparison correction. Only * for post hoc test are shown, *p < 0.05, **p < 0.01, ***p < 0.001

To understand how perturbations to neuronal cultures modulate network dynamics in HD, we increased neuronal activity by blocking GABA_A_ receptors with the GABA_A_ receptor antagonist bicuculline (60 μM, BIC). At the network level, disinhibition boosted coordinated activity throughout the cultures (Fig. 3a) and strongly increased the number of active neurons (BIC effect; *F*(1, 19) = 240.3, *p* < 0.001; Fig. 3b). When analyzing the impact of disinhibition specifically on coordinated activity, we found an increase in the percentage of neurons participating in network bursts in both genotypes (BIC effect; *F*(1, 7) = 42.34, *p* < 0.001; Fig. 3c). Although BIC application increased the network IBI in both WT and HD cultures (BIC effect; *F*(1, 7) = 21.09, *p* = 0.02; genotype-BIC interaction effect; *F*(1, 7) = 11.28, *p* = 0.01; Fig. 3d), the Bonferroni post hoc tests revealed significant treatment effect only in WT cultures (*p* = 0.001). Regarding the internal properties of network bursts, both genotypes showed a significant increase in the number of spikes per neuron and burst duration (Additional file 6: Figure S6). Moreover, activity rate of individual neurons increased upon disinhibition across genotypes (Additional file 7: Figure S7).

The effect of disinhibition on global activity rate and the percentage of independent spikes was also evaluated. As expected, global activity rate increased after the addition of BIC in striatal cultures in WT and HD, according to higher levels of activity (BIC effect; *F*(1, 19) = 67.6, *p* < 0.001; Fig. 3e). On the other hand, the percentage of independent spikes decreased in both genotypes, in agreement with a higher correlated activity (BIC effect; *F*(1, 19) = 134.8, *p* < 0.001; Fig. 3f).

These results show that blockade of GABA_A_ receptors induces strong coordinated burst formation. The effects generated by BIC are similar across genotypes in striatal cultures. Although the non-significant increase of the IBI in HD could suggest a differential effect depending on the genotype, both genotypes reach similar values after complete blockade of the inhibition. This indicates that the remaining excitatory component in the cultures, which are responsible for orchestrating activity, is comparable in both WT and HD cultures. Therefore, the increased striatal inhibitory activity observed in HD [10, 13] may be responsible of the alterations in coordinated ensemble activity observed under basal conditions, which could underlie information processing deficits within the basal ganglia network in HD.

### Blockade of GABA_A_ receptors differently assembles network topology in WT and HD striatal cultures

**Fig. 4:**
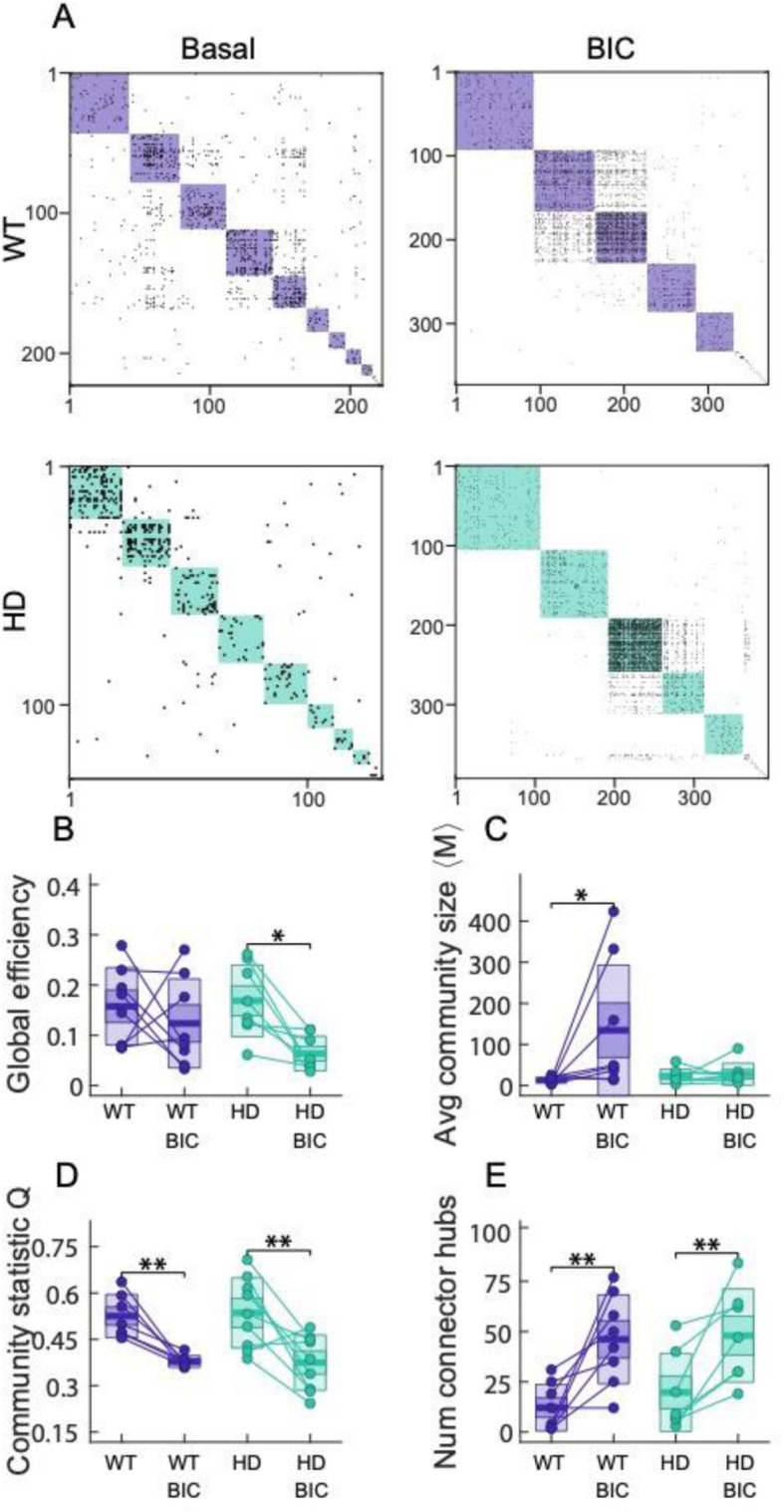
Disinhibition shapes different network properties in WT or HD mouse models. **a** Representative examples of WT and HD striatal effective connectivity matrices after disinhibition with BIC 60 μM. Color boxes highlight communities. **b**–**e** Change in network properties for cultures before and after disinhibition. **b** Global efficiency, **c** average community size, **d** community statistic *Q*, and **e** average number of connector hubs. Each circle represents a single experiment (STR WT *n* = 8; STR HD *n* = 9; CTX WT *n* = 19; CTX HD *n* = 22), thick line the mean, thick shaded area the standard error of the mean, and thin shaded area the standard deviation. Statistical analysis was performed using mixed ANOVA and posterior post hoc test with Bonferroni’s multiple comparison correction. Only * for the post hoc test are shown, *p < 0.05, **p < 0.01, ***p < 0.001

We evaluated how the network topology of striatal primary cultures from WT and HD mice is reorganized after BIC application (Fig. 4). Global efficiency (Fig. 4b) was reduced in striatal cultures (BIC effect; STR: *F*(1, 15) = 6.4, *p* = 0.02). However, post hoc analysis showed significant treatment effect only on HD cultures (*p* = 0.018). The decrease in global efficiency reflects that most neurons within the culture are effectively connected after disinhibition, but the strongest connections detected by Generalized Transfer Entropy tend to cluster in independent communities, resulting in a decreased efficiency of the network. Average community size analysis (Fig. 4c) showed treatment effect. Community size increased (*F*(1, 15) = 4.7, *p* = 0.04) and post hoc analysis detected significant treatment effect in WT (*p* = 0.01). The finding that community size only increased in WT striatal cultures indicates a higher capacity of recruitment of active neurons to the community. This suggests that HD striatal neurons might not be able to engage new neurons to enlarge neuronal communities. Moreover, the community statistic *Q* (Fig. 4d) decreased in WT and HD networks (BIC effect; *F*(1, 15) = 29.74, *p* < 0.001) and the number of connector hubs (Fig. 4e) increased after disinhibition (BIC effect; *F*(1, 15) = 34.76, *p* < 0.001), demonstrating an increase in the proportion of inter-modular connections.

Complete disinhibition of the cultures produced a dramatic change on the network properties. Striatal network disinhibition generated networks with increased community size and a higher number of connector hubs, which in turn increased inter-modular connections and reduced *Q*. Despite the increased neuronal activity and network reorganization, the global efficiency of the network was maintained in WT cultures. Conversely, HD cultures were unable to recruit neurons to increase the size of its communities, and overall, global efficiency was reduced after blockade of GABA_A_ receptors, highlighting altered response of the HD network to system disinhibition.

### NMDA enhances global activity in HD striatal primary cultures

**Fig. 5:**
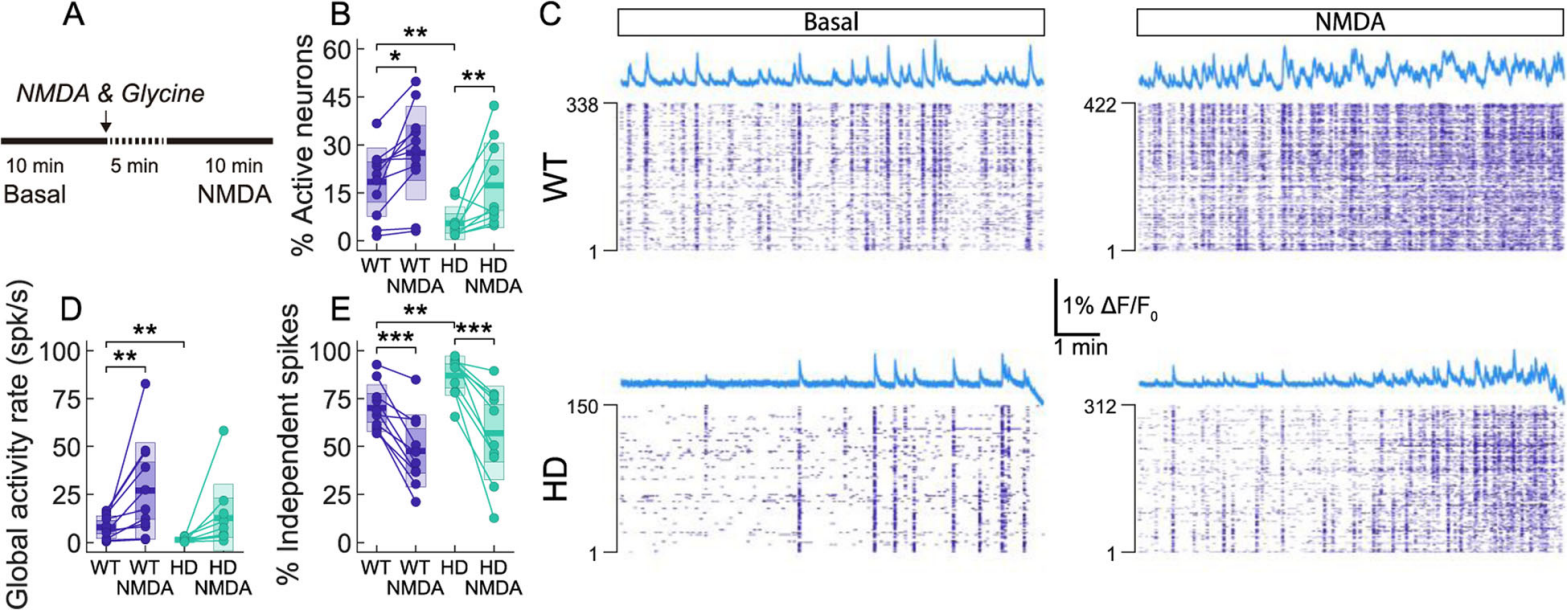
NMDA rises global neuronal activity in WT and HD striatal primary cultures. **a** Experiment timeline. Spontaneous neuronal activity was recorded during 10 min in basal conditions and after addition of 10 μM NMDA + 10 μM glycine. **b** Percentage of active neurons. **c** Representative raster plots of spikes and average fluorescence trace before (left) and after (right) addition of NMDA for WT (upper) and HD (lower). **d** Global activity rate of the cultures and **e** fraction of independent spikes. Each circle represents a single experiment (STR WT *n* = 10; STR HD *n* = 10), thick line the mean, thick shaded area the standard error of the mean, and thin shaded area the standard deviation. Statistical analysis was performed using mixed ANOVA and posterior post hoc test with Bonferroni’s multiple comparison correction. Only * for the post hoc test are shown, *p < 0.05, **p < 0.01, ***p < 0.001

To further understand how striatal network self-organize upon increased neuronal activation, we mimicked an increase of excitatory drive in striatal cultures by boosting the glutamatergic system through an increase in the extracellular NMDA concentration (Fig. 5a). In our experiments, 10 μM NMDA application in striatal cultures led to an increase in the percentage of active neurons (NMDA effect; *F*(1, 20) = 20.59, *p* < 0.001; Fig. 5b) and an overall significant increase in individual neuronal activity (Additional file 8: Figure S8). Cultures became globally more active, but network bursts were undefined and highly variable (Fig. 5c). These activity patterns were also observed in other culture preparations and are associated with excitation-inhibition levels and critical dynamics [33].

Due to poor network burst detection after NMDA addition, we only evaluated global network activity through global activity rate and independent spikes (Fig. 5d, e). Global activity rate significantly increased after addition of NMDA (NMDA effect; *F*(1, 18) = 5.925, *p* = 0.002; Fig. 5d), but a post hoc test showed differences only after treatment in WT (*p* = 0.005), while genotype differences were only present under basal conditions (*p* = 0.002). On the other hand, the fraction of independent spikes within the system (Fig. 5e) was significantly decreased in striatal cultures after the addition of NMDA (NMDA effect; *F*(1, 18) = 11.83, *p* < 0.001). Post hoc analysis revealed that NMDA decrease independent spikes in both genotypes, and genotype differences only existed in basal conditions (*p* = 0.004), not after NMDA treatment. Interestingly, increased activity by NMDA attained levels in HD cultures was similar to that observed in WT in baseline conditions, suggesting that boosting excitatory activity could facilitate information flow throughout the HD striatal neuronal network.

### Boosting excitatory drive with NMDA partially restores network topology in HD striatal primary cultures

**Fig. 6:**
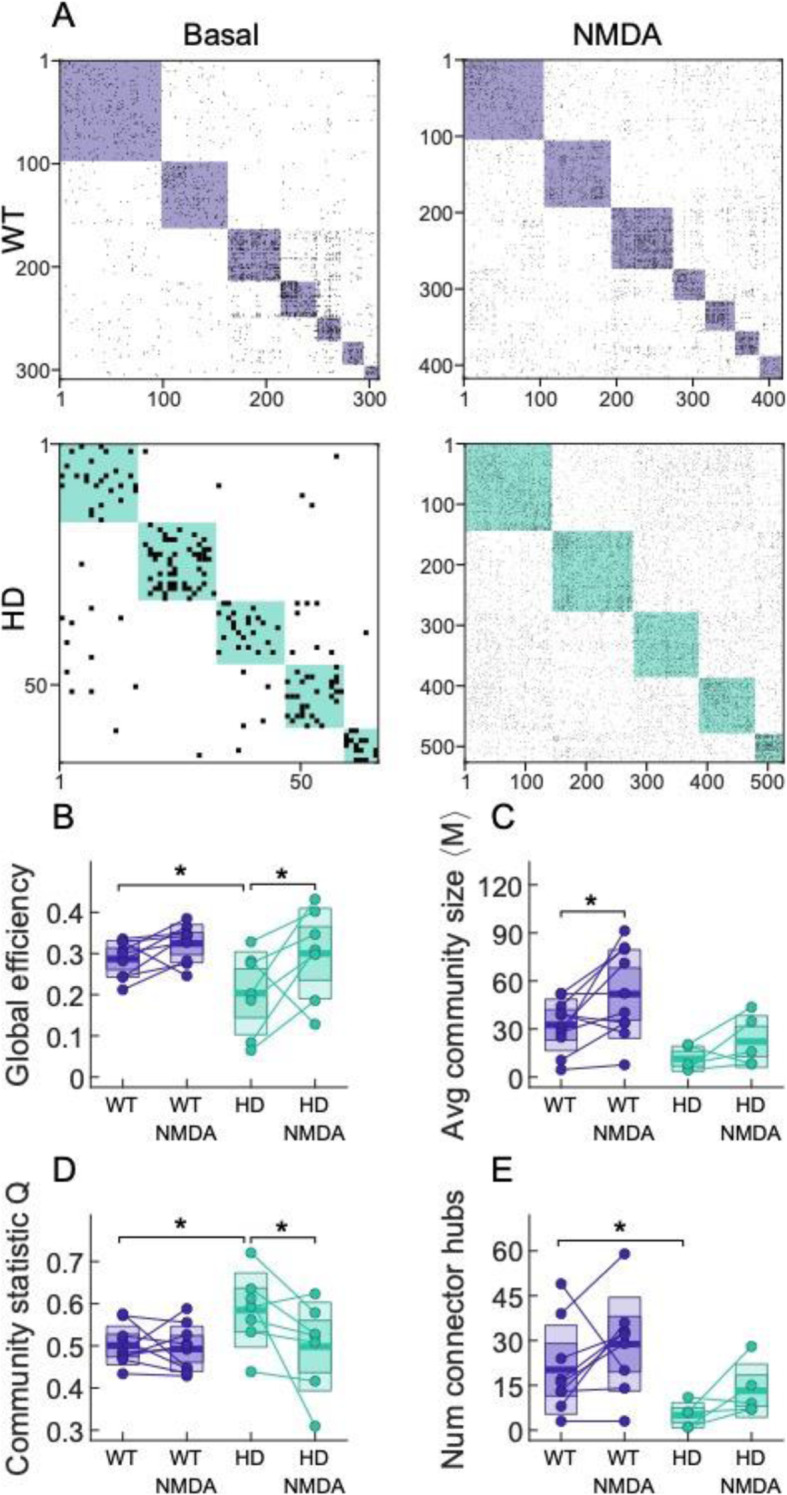
NMDA treatment restores neuronal network topology in striatal HD cultures. **a** Representative examples of WT and HD striatal effective connectivity matrices after NMDA 10 μM. Color boxes highlight communities. **b**–**e** Change in network properties for cultures before and after NMDA treatment. **b** Global efficiency, **c** average community size, **d** community statistic Q, and **e** average number of connector hubs. Each circle represents a single experiment (STR WT *n* = 10; STR HD *n* = 7), thick line the mean, thick shaded area the standard error of the mean, and thin shaded area the standard deviation. Statistical analysis was performed using mixed ANOVA and posterior post hoc test with Bonferroni’s multiple comparison correction. Only * for the post hoc test are shown, *p < 0.05, **p < 0.01, ***p < 0.001

The glutamate-induced increase in neuronal activity reorganized the network topology differentially in WT than in HD cultures (Fig. 6). Global efficiency was increased after NMDA (NMDA effect; *F*(1, 15) = 8.301, *p* = 0.01). However, post hoc analyses revealed significant NMDA effects only in HD cultures (*p* = 0.01), while genotype differences were present only in basal conditions (*p* = 0.04) and disappeared after NMDA treatment. NMDA treatment modulated the size of the communities (NMDA effect; *F*(1, 15) = 15.44, *p* = 0.016) (Fig. 6b); however, a post hoc test showed a significant increase of the community size only in WT cultures (*p* = 0.016). The community statistic *Q* was also modulated by NMDA (NMDA effect; *F*(1, 15) = 5.273, *p* = 0.04), although post hoc test showed a decrease in statistic *Q* only in HD cultures, suggesting an increase in inter-modular connections specifically in HD while losing genotype differences found before treatment (*p* = 0.017). Regarding the number of connector hubs, there was no significant treatment effect, but significant genotype differences were only present under basal conditions (*p* = 0.05) (Fig. 6d).

Thus, NMDA treatment increased community size without affecting the other network topology observables in WT cultures. In HD, effective connectivity analysis revealed that NMDA restored most network observables, which reached values comparable to WT cultures under basal conditions. These results hint at an improvement of information flow throughout the neuronal network by increasing excitatory drive in the HD striatal system.

## Discussion

The combination of large-scale calcium imaging recordings and network dynamics in isolated cultures provides unprecedented insight about the nature of HD pathology at the network level. The presence of mHtt in striatal neurons leads to defective ensemble activity and network topology, reflecting a neuronal self-organization that lessens signal transmission. Interestingly, coordinated ensemble activity and network topology are preserved in isolated cortical systems, despite the presence of mHtt. Moreover, our data shows that GABA_A_ receptor blockade modulates network coordinated activity throughout the cultures, while NMDA increases overall individual neuronal activity in detriment of network bursts. These effects generate reorganization of network topology without modulating the global efficiency of the network. However, these perturbations differently affect WT and HD striatal cultures, highlighting the vulnerable nature of the network structure in the striatum of HD. Nonetheless, NMDA application partially rectifies network alterations in HD, suggesting that HD network dysfunction can be counterbalanced by increasing excitatory drive.

### Network-wide alterations in HD are intrinsic to the striatal system

Although the striatum is mainly composed by inhibitory neurons, we showed that isolated striatal cultures are functionally active. Some authors claimed that striatal in vitro cultures remain silent in the absence of cortical input [34]. However, we and others previously described active striatal cultures [23, 35], possibly owing to the availability of more sensitive detection methods and a higher number of simultaneously analyzed neurons.

Additionally, we detect a subset of striatal neurons displaying spontaneous coordinated activity in vitro measured as network bursts, indicating the presence of a functional network. In vivo studies showed that coordinated MSNs and interneuron activity in the striatum are essential for locomotion [36], which is tightly modulated by excitatory and inhibitory activity. Indeed, a lack of MSN inhibition or increased MSN activity by cholinergic modulation induces MSN synchronization [36, 37], which prevents cortical inputs to select among different cell assemblies, thus curtailing movement initiation. Specifically, blockade of GABA_A_ receptors increased the peaks of synchronous activity in the striatum and recruited more cells during synchrony peaks [37], akin to the effects of BIC observed in our striatal system. Despite the fundamentally different nature of the in vivo and in vitro networks, the reduced striatal ensemble activity in basal conditions together with its inability to self-organize upon disinhibition might be a common mechanism of HD striatal network alteration in HD.

On the other hand, pharmacological application of NMDA in our isolated striatal cultures increased the number of active neurons and their individual spiking rate, although it decreased correlated activity. Under different experimental conditions, NMDA application in striatal slices [37] or in cortico-striatal microfluidic chambers [38] led to an increase of correlated activity. These observations, together with our results, suggest that coordinated activity in cortico-striatal circuits emerges due to a synchronous cortical excitatory drive and not as an intrinsic NMDA receptor activation in the striatum.

Although both WT and HD cultures showed spontaneous individual and coordinated activity, we observed a decreased number of active neurons specifically in the HD striatal system, in agreement with previous findings [23]. Remarkably, our results show that striatal—but not cortical—network burst patterns are altered in the HD striatal system. The finding that network burst properties (i.e., burst duration and amplitude) are not different, but their frequency is decreased, suggests that striatal HD cultures have a reduced capacity for burst generation, at least in part, due to deficient activity recruitment of neighboring neurons, which does not occur in the cortex. This decreased capacity reveals a dysfunctional connectivity of the striatal HD network. Similar results were shown in previous studies from in vivo electrophysiological recordings in freely moving HD mice models [39] and brain slices from HD models (Cepeda et al. [10]). In both cases, a decreased number of connected pairs of MSNs as well as a reduced correlation among striatal neurons were consistently reported [10, 39].

Hence, our findings demonstrate that network-wide alterations are intrinsic to the striatal system. We provide evidence that striatal circuits have a decreased capacity to transmit information and are more segregated, while individual neuronal activity is preserved. Our data in isolated cultures support the idea that fundamental striatal network deficiencies in HD might be attributed to cell-autonomous mechanism of mHtt within the striatum [40], in addition to the described aberrant striatal afferences [9, 38].

### Altered excitation/inhibition balance within the striatum shapes HD network dysfunction

Within the striatal network, decreased network bursting activity could be related to two possibilities: insufficient excitation within the system or aberrant inhibition. In the absence of external inputs, as the case of our striatal cultures, the reduction of coordinated activity must arise from an intrinsic striatal mechanism. Indeed, local GABAergic neurotransmission within the striatum strongly shapes the temporal processing of cortical inputs [41]. Some authors suggested that striatal aberrant inhibition might account for a compensatory mechanism aimed to counteract the increased glutamatergic neurotransmission coming from the cortico-striatal afferences [42]. In our work, the isolated striatal cultures showed network alterations without the presence of cortical inputs, suggesting that altered GABAergic neurotransmission is a core feature of HD.

Previous results have reported increased inhibition onto striatal MSNs in different ex vivo models of HD [10]. These alterations in GABAergic currents reflect abnormal synaptic transmission locally in the striatum and point to striatal feedforward inhibition as a possible source of the increased GABAergic transmission, for instance through GABAergic interneurons or through striatal feedback inhibition from other MSNs via axon collaterals. Indeed, somatostatin-positive GABAergic interneurons within the striatum elicit higher levels of spontaneous activity in R6/2 models [10]. Additionally, HD parvalbumin-positive GABAergic interneurons evoke larger amplitude responses in MSNs to which are connected upon selective optogenetic stimulation [10]. Interestingly, our in vitro data support the idea that increased local inhibition might be the origin of the striatal network dysfunction in HD.

### Increasing the excitatory drive enhances global activity in HD

The cerebral cortex and striatum are part of a richly interconnected network involved in motor control. It is not possible to affect one region of this network without impacting the others [43]. Indeed, previous studies reported that normalization of cortical activity with the selective reduction of mHtt expression in the cortex partially ameliorates motor impairment and psychiatric-like disturbances in vivo [44] and some features of striatal activity in vitro [9]. These reports highlight the fact that restoration of one element of the circuit could compensate some neuropathological features [1].

Our results agree with the above concepts, as pharmacological application of NMDA increases communication throughout the neuronal network specifically in HD striatal cultures, as reflected by a decrease in the proportion of isolated spikes. Interestingly, evaluation of functional connectivity in these cultures showed a strengthening in functional integration, which resulted from a selective increase of the global efficiency together with a higher proportion of connections between communities. Thus, NMDA application decreased network segregation in HD striatal cultures. However, despite recovery of some measurements of neuronal communication and network topology, NMDA application results in an increased, non-coordinated individual activity. Therefore, finding new strategies to increase the excitatory drive within the HD striatum emerges as an attractive therapeutic approach for further exploration.

Taking into account that NMDA signaling promotes neurodegeneration by excitotoxic damage in HD MSNs [45], it might seem counterintuitive that an increase in NMDA enhances network performance. In fact, amantadine, a non-competitive NMDA antagonist, is the suggested alternative for tetrabenzine for the pharmacological treatment of chorea in HD, based on small clinical trials [46]. However, the efficacy of amantadine is controversial, as it is overall not effective in reducing chorea in HD and may even worsen cognitive and psychiatric alterations, highlighting the deleterious effects of antagonizing NMDARs [47]. Due to the highly challenging task of manipulating NMDARs, an increment of excitatory drive through other receptors should be explored.

In summary, this work highlights the study of coordinated ensemble activity and network topology in cultures as a valuable tool to decipher the alterations in the basal ganglia network. We described a more segregated network in the striatum of HD, which results in defective network integration capacity due to increased intra-striatal inhibition. Our results further suggest that an increase in excitatory drive over the striatal HD network compensates network deficits and improves the integration/segregation balance, therefore increasing the information processing capacity of the basal ganglia circuits in HD. Thus, exploring targets that increase excitatory drive in striatal circuits and that rebalance individual and collective activity might uncover novel therapeutic interventions for HD.

## Conclusions

We unfolded key deficiencies in the network activity of the HD striatal cultures, which translate into a loss in communication and integration capacities. Our work reveals the importance of the study of the neuronal collective dynamics rather than individual neuronal dysfunction in a neurodegenerative context. Our framework, with a comprehensive study of culture dynamics, provides a valuable tool to evaluate network dysfunction in neurodegenerative disorders.

## Methods

### Animals

R6/1 transgenic mice expressing exon-1 of mutant huntingtin were acquired from the Jackson Laboratory (Bar Harbor, ME, USA) and maintained in a B6CBA background. Mice genotype was obtained as previously described [48]. Wild-type (WT) littermates were used as the control group. Animals were housed in a room kept at 19–22 °C and 40–60% humidity under a 12:12-h light/dark cycle with access to water and food ad libitum.

### Primary cultures

Cortical and striatal primary cultures were performed as previously described [23]. Brains from E.18 WT and HD embryos were excised and placed in Neurobasal medium (21103-049, Gibco). The striatum and cortex were dissected and gently dissociated with a fire-polished glass Pasteur pipette. Cells were seeded onto 12-mm glass coverslips pre-coated with 0.1 mg/ml poly-D-lysine (P0899, Sigma) at a density of 80,000 cells/cm^2^. Neurobasal medium supplemented with Glutamax (35050-038, Gibco) and B27 (17504-044, Gibco) was used to grow cells in serum-free conditions. Cultures were maintained at 37 °C in a humified atmosphere containing 5% CO_2_ until 15 days in vitro (DIV). Primary cultures were produced individually from each embryo in a genotype-blind manner. Tissue samples from the embryo tail was used for DNA extraction, and presence/absence of mHtt was determined by PCR amplification as previously described [49].

Experiments were performed in cultures coming from a minimum of three different litters. To control any source of variability, all the cultures were seeded at the same embryonic day (E18.5), measurements were carried out at the same day in vitro (15 DIV), and reagents used were from the same batch.

### Immunofluorescence

Striatal primary cultures grown on glass coverslips were fixed at 15 DIV with 4% paraformaldehyde (PFA) for 10 min, washed with PBS, and incubated with 0.1 M glycine-PBS for 15 min. Coverslips were rinsed three times with PBS and incubated during 1 h at RT with blocking solution containing 0.3% Triton X-100 and 1% bovine serum albumin (BSA) in PBS. Then, coverslips were incubated at 4 °C overnight with blocking solution containing the following primary antibodies: MAP2 (Sigma, 1:1000) and GFAP (Dako, 1:1000). The remaining primary antibody was removed with three washes with PBS, and coverslips were incubated during 1 h at RT with Alexa488 donkey anti-mouse and Cyanine 3 donkey anti-rabbit (1:150; Jackson ImmunoResearch, West Grove, PA) secondary antibodies. Finally, coverslips were mounted on microscope slides with DAPI Fluoromount-G (SouthernBiotec).

### Image analysis

Striatal neuron staining was obtained with an inverted microscope (Leica DMI6000 B) equipped with a Hamamatsu Orca-ER camera and an external light source for fluorescence excitation (Leica CTR6000). A mosaic containing 10 × 13 images (385.24 × 293.52 μm) with 10% overlapping between images with a total surface of 1,2 mm^2^ (3610.12 × 3344.69 μm) was analyzed. Mosaics from two different embryos for genotype (WT and R6/1) from three different litters were processed. Number of GFAP- and MAP2-positive cells, quantification of branches, and neurite length from MAP2 immunofluorescence images were performed using Cell Profiler v2.8 software as previously described [23, 50].

### Calcium imaging recordings

Calcium fluorescence recordings were carried out at 15 DIV using the cell-permeant calcium sensitive dye Fluo4-Acetoxymethyl ester (Fluo4-AM) (Invitrogen, Thermo Fisher), similar to [23]. Fluo4 1 mM dissolved in DMSO was added to the culture medium to a final concentration of 1 μM and incubated for 20 min at 37 °C. After incubation, cultures were placed in a 35-mm-diameter glass bottom chamber (P35G-0-14-C; MatTek Corporation) for recording. Recordings were carried out in pH-stable (7.4) external medium (EM). EM consisted of HEPES (4-(2-hydroxyethyl)-1-piperazineethanesulfonic acid), 10 mM; NaCl, 128 mM; KCl, 4 mM; glucose, 10 mM; sucrose, 45 mM; CaCl_2_, 2 mM; and MgCl_2_, 1 mM. The recording chamber was mounted on an Olympus IX70 inverted microscope equipped with a Hamamatsu Orca Flash 4.0 V2 (Digital CMOS camera C11440-22CU) camera and Dual OptoLED power supply (Cairn Research Ltd) as a source of light. Fluorescence images were acquired at 20 frames per second (fps) at room temperature (RT) with a × 5 objective.

### Calcium imaging analysis

Recordings were first time-averaged across the whole recording to obtain a clear image of the cell bodies.

#### ROI detection

Cells were automatically detected as regions of interest (ROIs) using the time-averaged image as follows. Background noise was removed by first normalizing the image between its 20% and 30% percentile. From the normalized image, a Gaussian filter of 7 pixels width was applied and subtracted from the normalized image. The resulting image was then binarized using a threshold of 0.08 (the threshold value was chosen visually to maximize the detection of cell bodies). Any holes in the resulting image were then filled. Next, we performed a morphological opening with a square structuring element of size 4. Finally, any connected component with less than 10 pixels was removed. The remaining set of connected components defined the ROI set.

#### Trace extraction and baseline correction

Traces were extracted for each ROI by spatially averaging between its pixels across the whole recording. Each trace was then smoothed by applying a square sliding window 5 frames wide. Baseline was estimated by taking a reference point each 50 s block, and its position within the block determined by the value closest to the 10% lower fluorescence percentile within that block. A smoothing spline was then fitted using this point set. This baseline was subtracted from the original trace and finally normalized to 100 × (*F* − *F*_0_)/*F*_0_ relative units.

#### Trace classification

The resulting traces were classified into three groups (neuron, glia, and silent) using a supervised machine learning approach. First, traces were visually inspected and a subset belonging to each class was manually selected. Then, an ensemble classifier was trained with this subset using adaptive boosting. The classifier uses a set of features obtained from the statistical moments of the traces (average, variance, skewness, etc., see [28] for details). The resulting classification was visually inspected and refined by changing the training set as needed.

#### Spike train reconstruction from calcium transients

Spikes from neurons were inferred using a modified Peeling method [51] that skips the last precise-timing pattern-matching step. For the underlying calcium model, a single decaying exponential with non-saturating dynamics with amplitude 1% ∆*F*/*F*_0_ and decay 3 s was used on all experiments.

#### Network burst detection

Network bursts, i.e., large ensembles of coordinated neuronal activity, were detected by first obtaining a smoothed signal of active cell, i.e., on each frame, we computed the number of unique neuronal activations (reconstructed spikes) on the previous 1-s interval. Putative network bursts were identified using a Schmitt trigger on that signal with upper and lower thresholds of 10% and 5% active neurons, respectively. Finally, any burst pairs that were less than 0.5 s apart were merged and bursts with less than 10 participating neurons were discarded. Network bursts were defined as the remaining set. A culture was deemed bursty if its inter-burst interval (IBI) was below 45 s. The 45-s mark was chosen based on posterior pharmacological experiments. After bicuculine treatment, cultures with an IBI below 45 s showed an increase in IBI. Cultures with an IBI above 45 s showed a decrease in IBI. IBI separation mark was verified via blind *k*-means clustering separation of IBI changes.

#### Network inference

Effective network topology was estimated using a modified version of the Generalized Transfer Entropy (GTE) framework previously described in [52]. In short, for any two-time series *I* and *J*, an effective connection is established from *I* to *J*, when the information contained in *I* significantly adds predictive power on inferring future states of *J*, i.e., there is a causal relation in the Granger sense. For the input data, we used the actual spike trains instead of the derivative of the fluorescence signal. Instant feedback was present, and Markov Order 2 was used. For any connection *I* to *J*, significance was established by comparing the actual GTE estimate with those from the joint distribution of all input *X* to *J* and output *I* to *Y* scores (for any *X* and *Y*). A connection was deemed significant if it exceeded the mean + 2 standard deviations of the joint distribution. Standard network measures were computed using the Brain Connectivity Toolbox [53]. Network modules were detected using a fast implementation of Louvain’s algorithm on the largest connected component. Network inference was only performed on cultures with more than 25 active neurons.

### Code accessibility

Analysis of calcium imaging recordings was performed using NETCAL, a software package that was developed on MATLAB for large-scale analysis of calcium imaging experiments in neuronal cultures. The software is described in [28] and can be downloaded in www.itsnetcal.com.

### Pharmacological modulation of primary cultures

Spontaneous activity was initially recorded for 10 min. In a subset of experiments, GABA_A_ receptor-antagonist BIC methiodide (Sigma-Aldrich, MO, USA) was added to the culture to a final saturation concentration of 60 μM [38, 54]. After a 5-min stabilization period, spontaneous activity from the same field of view was recorded for an additional 10 min. The same procedure was followed for NMDA experiments. Different NMDA doses were initially tested (1, 10, 50, 100 μM), and a dose of 10 μM NMDA was finally selected as higher doses increased baseline fluorescence levels that masked the measurements of neuronal activity at the single-cell level. NMDA was combined with 10 μM glycine (both from TOCRIS, Bristol, UK).

### Experimental design and statistical analysis

Each WT and R6/1 culture was obtained from individual embryos from the same litter. A minimum of three different litters were used for each experiment to avoid litter bias. MATLAB (MathWorks, Natick, MA) was used to perform statistical analysis. To assess genotype alterations in basal conditions, statistical analysis was performed using unpaired two-sample Student’s *t* test. To assess changes after bicuculline or NMDA treatment, differences between genotype, treatment, and genotype-treatment interaction were analyzed using mixed-effect ANOVA and followed by the Bonferroni post hoc test when appropriate. Values of *p* < 0.05 were considered statistically significant, and its exact value reported (*p* values lower than 0.001 were reported as *p* < 0.001). Outliers were automatically excluded following the quartile criteria. The exact sample size (*n*) for each experimental group is provided in the figure legends.

## Supplementary information

**Additional file 1: Figure S1.** Characterization of the striatal primary cultures. (**A**) Representative epifluorescence microscopy images showing nuclei stained with DAPI (blue), neurons stained with MAP2 (green), astrocytes stained with GFAP (red) and the merge of the three channels. (**B**) Quantification of the neurons and astrocytes as percentage of positive cells in the cultures. (**C**) Quantification of the number of MAP2 immunolabelled branches per neuron and (**D**) total tree length in WT and HD striatal cultures. Data are presented as mean ± SEM (STR WT *n* =6, STR HD n =6). Scale bar, 50μm.

**Additional file 2: Figure S2.** Scheme of the high-throughput calcium imaging recording and analyses in primary cultures. (**A**) Representative average image of a striatal primary culture at 15 DIV. (**B**) Calcium fluorescence traces from individual neurons highlighted in (**A**). Black vertical lines indicate reconstructed spikes. (**C**) Raster plot of spikes from ∼1000 neurons simultaneously recorded in the culture field of view. Each row represents an individual neuron. (**D**) Average fluorescence trace from all neurons (grey) with the detected network bursts highlighted in color. Scale bar 100 μM.

**Additional file 3: Figure S3.** Litter-grouped spontaneously active neurons and coordinated ensemble activity. The data is the same as Fig. 1 of the main text but, to evaluate intra- and inter-litter variability, the measurements are separated according to litters and for both WT and HD. The plotted data correspond to: (**A**) percentage of active neurons in the cultures (STR WT *n* =3, 4, 13, 14, 4; STR HD n =3, 3, 12, 12, 4; CTX WT *n* =2, 2, 6, 3, 3, 2; CTX HD n =3, 2, 6, 6, 2, 3) (**B**) percentage of active neurons that participate in spontaneous network burst (STR WT *n* =1, 0, 13, 7, 0; STR HD n =1, 1, 9, 5, 0; CTX WT n =3, 2, 6, 3, 3, 2; CTX HD n =3, 2, 6, 6, 1, 3), (**C**) average network inter-burst interval (IBI) (STR WT n =1, 0, 13, 8, 0; STR HD n =1, 0, 9, 5, 0; CTX WT n =3, 2, 6, 3, 2, 1; CTX HD n =3, 2, 6, 5, 2, 3), (**D**) global activity rate of the cultures (STR WT n =3, 4, 13, 14, 4; STR HD n =3, 3, 12, 12, 4; CTX WT n =3, 2, 5, 3, 3, 2; CTX HD n =3, 1, 6, 6, 1, 3) and (**E**) fraction of independent spikes (STR WT n =3, 4, 12, 13, 4; STR HD n =3, 3, 12, 14, 4; CTX WT n =3, 2, 6, 3, 1, 2; CTX HD n =3, 2, 6, 6, 2, 2). Each dot in the plot represents a single culture, and each column a different litter, thick line the mean, thick shaded area the standard error of the mean and thin shaded area the standard deviation.

**Additional file 4: Figure S4.** Network burst shape is preserved in HD striatal and cortical cultures. (**A**) Average duration of network bursts. (**B**) Burst amplitude measured in number of spikes per participating neurons in a burst (STR WT *n*=22; STR HD *n*=15; CTX WT *n*= 19; CTX HD n=22). Each dot represents a single experiment, thick line the mean, thick shaded area the Standard Error of the Mean and thin shaded area the standard deviation. Statistical analysis was performed using Student t-test.

**Additional file 5: Figure S5.** Quantification of individual spontaneous activity in WT and HD striatal and cortical primary cultures. (**A**-**E**) Single neuron statistics. (**A**) Activity rate, i.e., average spikes per second of each neuron. (**B**) Inter-spike interval (ISI), i.e., average time between two consecutive spikes. (**C**) Bursting rate, i.e., frequency of single-cell bursts. (**D**) Average inter-burst interval (IBI), average time between consecutive individual bursts (STR WT *n*=38; STR HD *n*=37; CTX WT n= 19; CTX HD n=22). Each dot represents a single experiment, thick line the mean, thick shaded area the Standard Error of the Mean and thin shaded area the standard deviation. Statistical analysis was performed using Student t-test. * *p*< 0.05, ** *p*<0.01 compared to WT.

**Additional file 6: Figure S6.** Quantification of network burst shape before and after bicuculline application. (**A**) Average duration of the network burst and (**B**) average number of spikes contained in burst (amplitude) (STR WT *n*=4; STR HD *n*=5). Each dot represents a single experiment, thick line the mean, thick shaded area the Standard Error of the Mean and thin shaded area the standard deviation. Statistical analysis was performed using mixed ANOVA and posterior Bonferroni’s post-hoc test. * p< 0.05, ** p<0.01 *** *p*<0.001.

**Additional file 7: Figure S7.** Individual neuronal activity increases after GABA_A_ receptor blockade with bicuculline (BIC) in striatal cultures**.** (**A**-**D**) Quantification of the changes in the single cell averaged spiking activity features in basal conditions and after the addiction of BIC: (**A**) Spiking rate, (**B**) ISI, (**C**) average frequency of bursting activity of individual neurons and (**D**) inter-burst interval (IBI) (STR WT *n* = 11; STR HD *n* = 10). Each dot represents a single experiment, thick line the mean, thick shaded area the Standard Error of the Mean and thin shaded area the standard deviation. Statistical analysis was performed using mixed ANOVA and posterior Bonferroni’s post-hoc test. * p< 0.05, ** p<0.01 *** p<0.001.

**Additional file 8: Figure S8.** Individual neuronal activity increases after NMDA application in striatal cultures. (**A**-**D**) Quantification of the changes in the single cell averaged spiking activity features before and after addition of 10 μM NMDA + 10 μM glycine for WT and HD. (**A**) Spiking rate, (**B**) inter-spike interval (ISI), (**C**) Frequency of individual bursting activity (bursting rate) and (**D**) individual inter-burst interval. (STR WT n=11; STR HD *n*=9-10). Each dot represents a single experiment, thick line the mean, thick shaded area the Standard Error of the Mean and thin shaded area the standard deviation. Statistical analysis was performed using mixed ANOVA and posterior Bonferroni’s post-hoc test. * p< 0.05, ** p<0.01 *** p<0.001.

## Data Availability

The datasets used and/or analyzed during the current study are available from the corresponding author on reasonable request.

## Abbreviations

HD: Huntington’s disease
WT: Wild type
mHtt: Mutant huntingtin
MSNs: Medium spiny neurons
IBI: Inter-burst interval
BIC: Bicuculline
