# Supplementary figures and images for: Deficits in coordinated neuronal activity and network topology are striatal hallmarks in Huntington’s disease
Source: BMC Biol. 2020 May 28;18:58. doi: 10.1186/s12915-020-00794-4 (PMC7254676; doi:10.1186/s12915-020-00794-4)

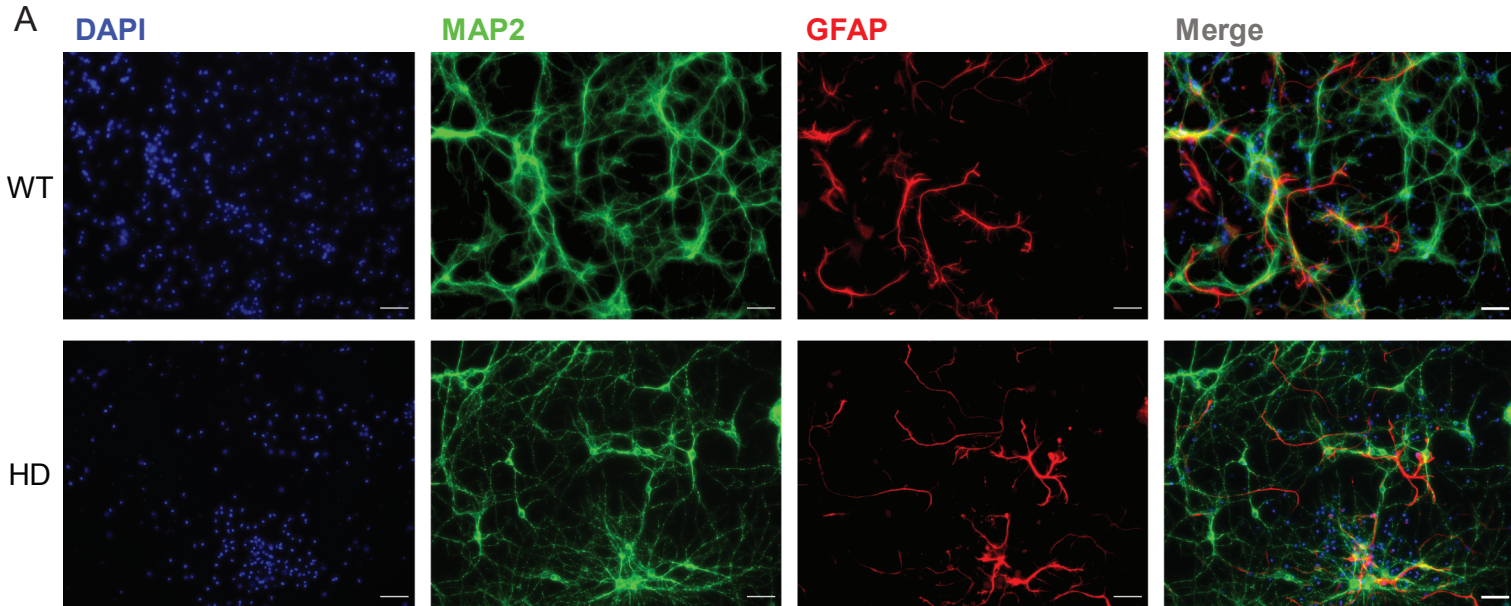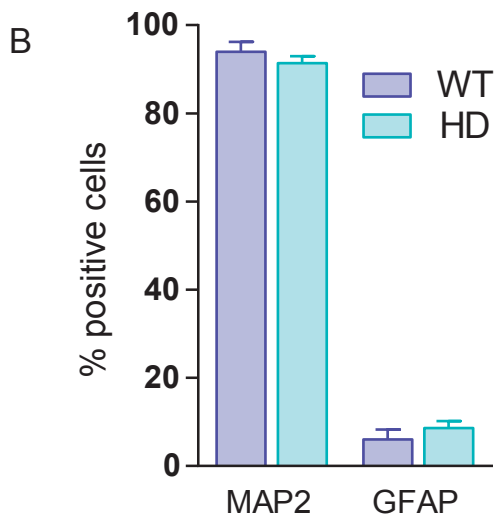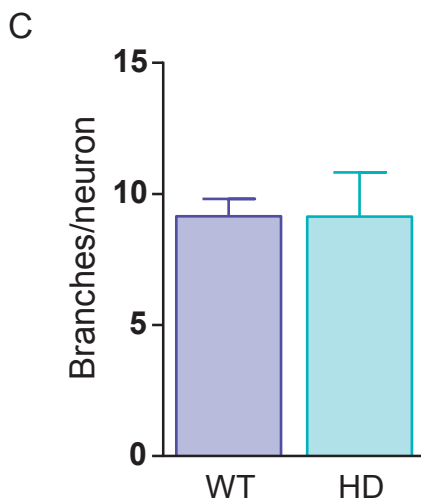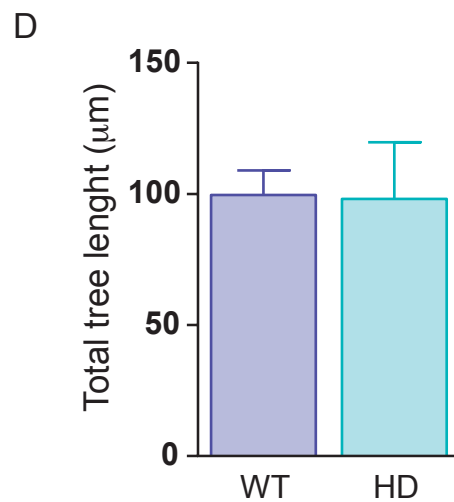

Supplement: Supplementary file 1 — Additional file 1: Figure S1. Characterization of the striatal primary cultures. (A) Representative epifluorescence microscopy images showing nuclei stained with DAPI (blue), neurons stained with MAP2 (green), astrocytes stained with GFAP (red) and the merge of the three channels. (B) Quantification of the neurons and astrocytes as percentage of positive cells in the cultures. (C) Quantification of the number of MAP2 immunolabelled branches per neuron and (D) total tree length in WT and HD striatal cultures. Data are presented as mean ± SEM (STR WT n =6, STR HD n =6). Scale bar, 50μm. [file 12915_2020_794_MOESM1_ESM.pdf]

A

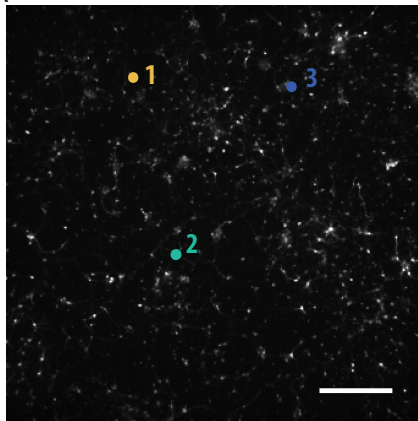

B

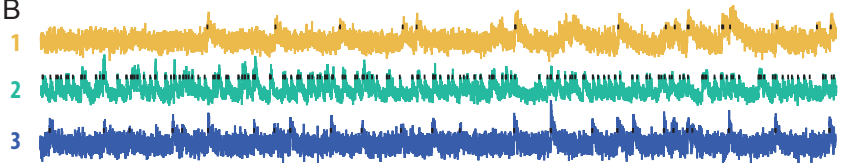

C

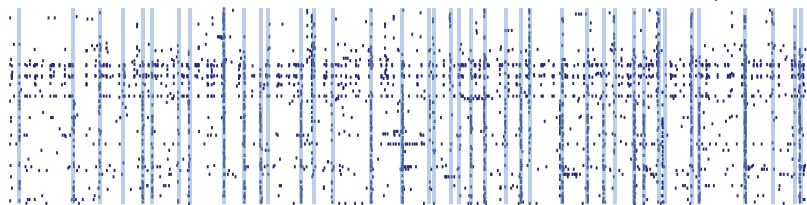

D

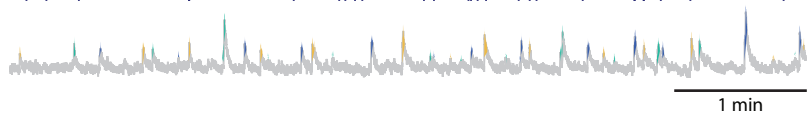

Supplement: Supplementary file 2 — Additional file 2: Figure S2. Scheme of the high-throughput calcium imaging recording and analyses in primary cultures. (A) Representative average image of a striatal primary culture at 15 DIV. (B) Calcium fluorescence traces from individual neurons highlighted in (A). Black vertical lines indicate reconstructed spikes. (C) Raster plot of spikes from ∼1000 neurons simultaneously recorded in the culture field of view. Each row represents an individual neuron. (D) Average fluorescence trace from all neurons (grey) with the detected network bursts highlighted in color. Scale bar 100 μM. [file 12915_2020_794_MOESM2_ESM.pdf]

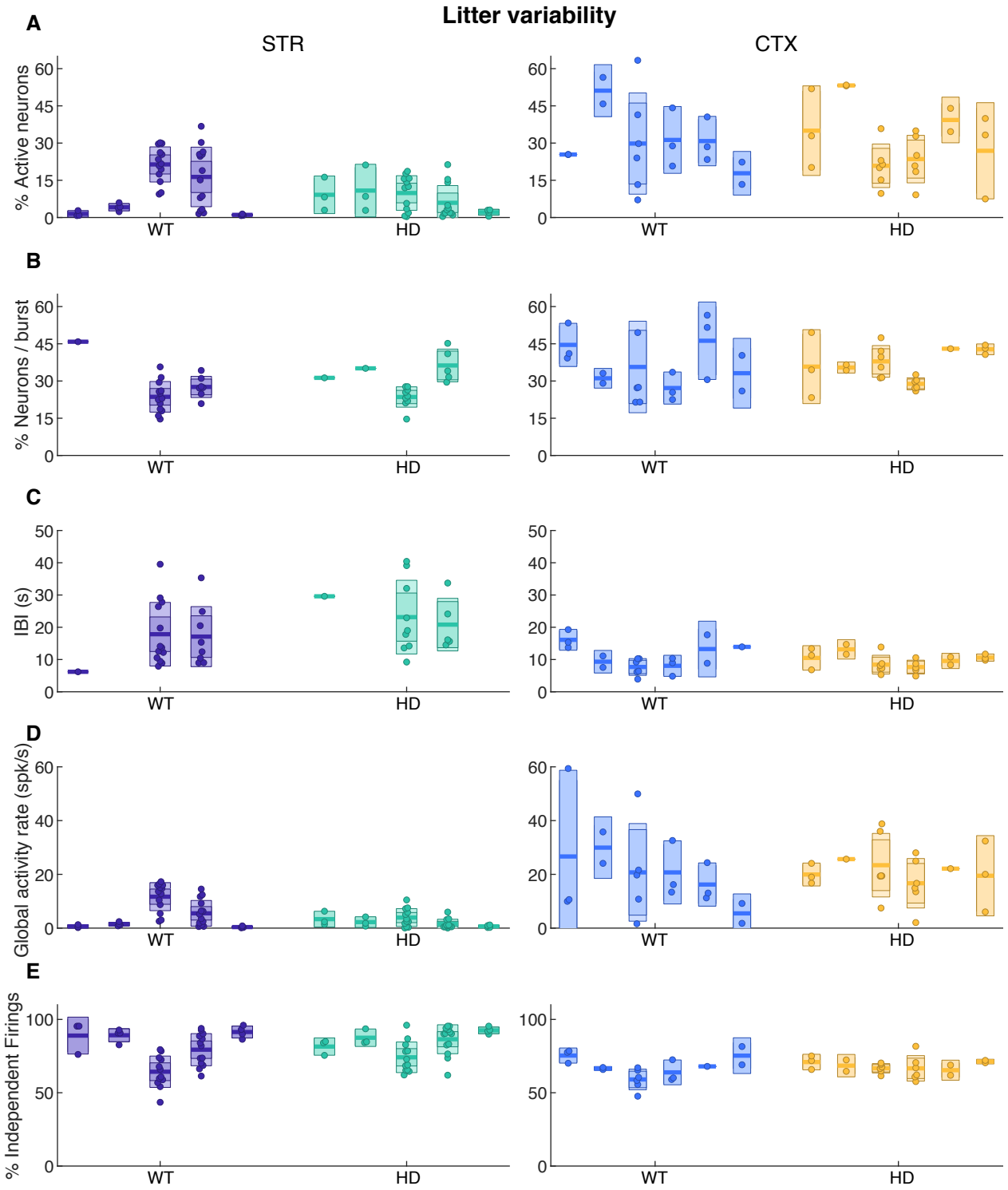

Supplement: Supplementary file 3 — Additional file 3: Figure S3. Litter-grouped spontaneously active neurons and coordinated ensemble activity. The data is the same as Fig. 1 of the main text but, to evaluate intra- and inter-litter variability, the measurements are separated according to litters and for both WT and HD. The plotted data correspond to: (A) percentage of active neurons in the cultures (STR WT n =3, 4, 13, 14, 4; STR HD n =3, 3, 12, 12, 4; CTX WT n =2, 2, 6, 3, 3, 2; CTX HD n =3, 2, 6, 6, 2, 3) (B) percentage of active neurons that participate in spontaneous network burst (STR WT n =1, 0, 13, 7, 0; STR HD n =1, 1, 9, 5, 0; CTX WT n =3, 2, 6, 3, 3, 2; CTX HD n =3, 2, 6, 6, 1, 3), (C) average network inter-burst interval (IBI) (STR WT n =1, 0, 13, 8, 0; STR HD n =1, 0, 9, 5, 0; CTX WT n =3, 2, 6, 3, 2, 1; CTX HD n =3, 2, 6, 5, 2, 3), (D) global activity rate of the cultures (STR WT n =3, 4, 13, 14, 4; STR HD n =3, 3, 12, 12, 4; CTX WT n =3, 2, 5, 3, 3, 2; CTX HD n =3, 1, 6, 6, 1, 3) and (E) fraction of independent spikes (STR WT n =3, 4, 12, 13, 4; STR HD n =3, 3, 12, 14, 4; CTX WT n =3, 2, 6, 3, 1, 2; CTX HD n =3, 2, 6, 6, 2, 2). Each dot in the plot represents a single culture, and each column a different litter, thick line the mean, thick shaded area the standard error of the mean and thin shaded area the standard deviation. [file 12915_2020_794_MOESM3_ESM.pdf]

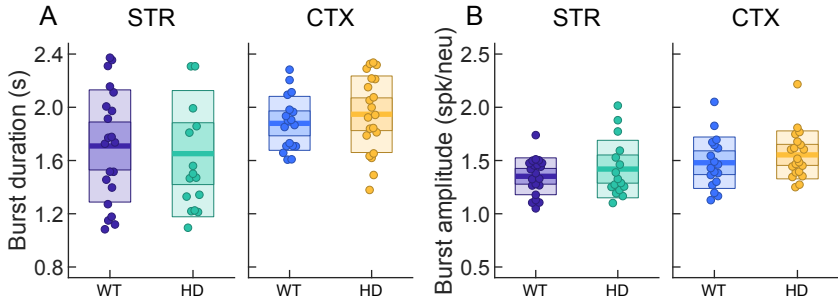

Supplement: Supplementary file 4 — Additional file 4: Figure S4. Network burst shape is preserved in HD striatal and cortical cultures. (A) Average duration of network bursts. (B) Burst amplitude measured in number of spikes per participating neurons in a burst (STR WT n=22; STR HD n=15; CTX WT n= 19; CTX HD n=22). Each dot represents a single experiment, thick line the mean, thick shaded area the Standard Error of the Mean and thin shaded area the standard deviation. Statistical analysis was performed using Student t-test. [file 12915_2020_794_MOESM4_ESM.pdf]

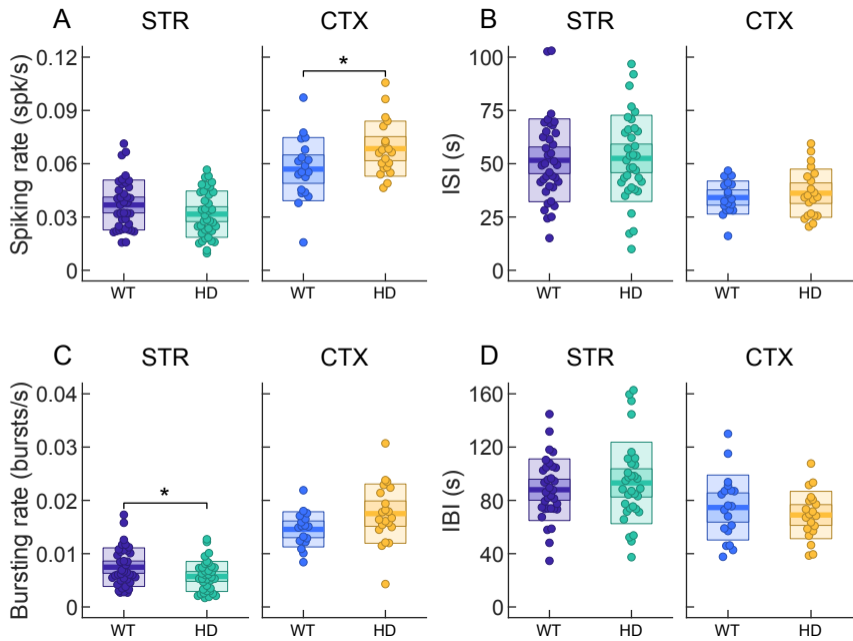

Supplement: Supplementary file 5 — Additional file 5: Figure S5. Quantification of individual spontaneous activity in WT and HD striatal and cortical primary cultures. (A-E) Single neuron statistics. (A) Activity rate, i.e., average spikes per second of each neuron. (B) Inter-spike interval (ISI), i.e., average time between two consecutive spikes. (C) Bursting rate, i.e., frequency of single-cell bursts. (D) Average inter-burst interval (IBI), average time between consecutive individual bursts (STR WT n=38; STR HD n=37; CTX WT n= 19; CTX HD n=22). Each dot represents a single experiment, thick line the mean, thick shaded area the Standard Error of the Mean and thin shaded area the standard deviation. Statistical analysis was performed using Student t-test. * p< 0.05, ** p<0.01 compared to WT. [file 12915_2020_794_MOESM5_ESM.pdf]

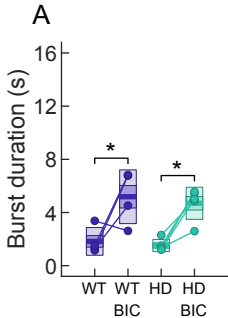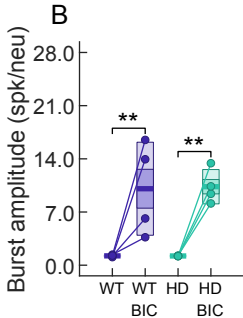

Supplement: Supplementary file 6 — Additional file 6: Figure S6. Quantification of network burst shape before and after bicuculline application. (A) Average duration of the network burst and (B) average number of spikes contained in burst (amplitude) (STR WT n=4; STR HD n=5). Each dot represents a single experiment, thick line the mean, thick shaded area the Standard Error of the Mean and thin shaded area the standard deviation. Statistical analysis was performed using mixed ANOVA and posterior Bonferroni’s post-hoc test. * p< 0.05, ** p<0.01 *** p<0.001. [file 12915_2020_794_MOESM6_ESM.pdf]

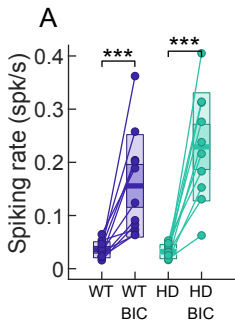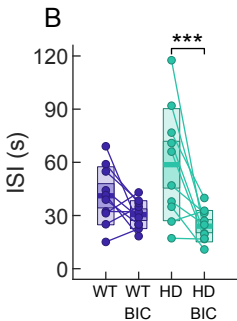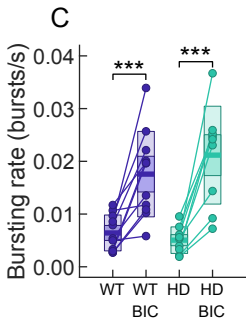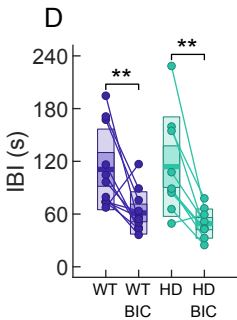

Supplement: Supplementary file 7 — Additional file 7: Figure S7. Individual neuronal activity increases after GABAA receptor blockade with bicuculline (BIC) in striatal cultures. (A-D) Quantification of the changes in the single cell averaged spiking activity features in basal conditions and after the addiction of BIC: (A) Spiking rate, (B) ISI, (C) average frequency of bursting activity of individual neurons and (D) inter-burst interval (IBI) (STR WT n = 11; STR HD n = 10). Each dot represents a single experiment, thick line the mean, thick shaded area the Standard Error of the Mean and thin shaded area the standard deviation. Statistical analysis was performed using mixed ANOVA and posterior Bonferroni’s post-hoc test. * p< 0.05, ** p<0.01 *** p<0.001. [file 12915_2020_794_MOESM7_ESM.pdf]

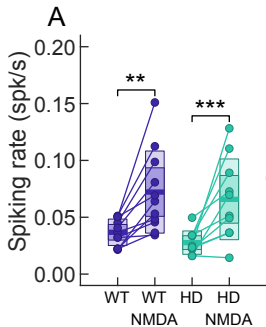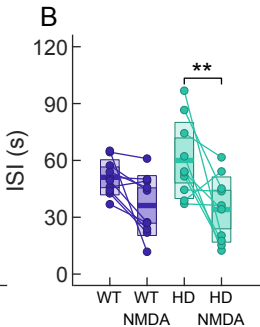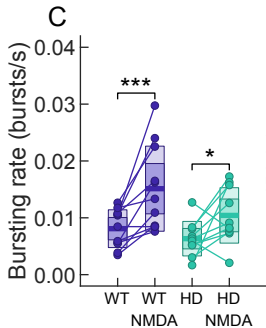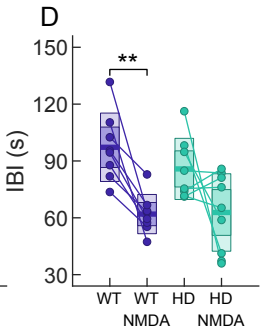

Supplement: Supplementary file 8 — Additional file 8: Figure S8. Individual neuronal activity increases after NMDA application in striatal cultures. (A-D) Quantification of the changes in the single cell averaged spiking activity features before and after addition of 10 μM NMDA + 10 μM glycine for WT and HD. (A) Spiking rate, (B) inter-spike interval (ISI), (C) Frequency of individual bursting activity (bursting rate) and (D) individual inter-burst interval. (STR WT n=11; STR HD n=9-10). Each dot represents a single experiment, thick line the mean, thick shaded area the Standard Error of the Mean and thin shaded area the standard deviation. Statistical analysis was performed using mixed ANOVA and posterior Bonferroni’s post-hoc test. * p< 0.05, ** p<0.01 *** p<0.001. [file 12915_2020_794_MOESM8_ESM.pdf]
